# Novelty N2-P3a Complex and Theta Oscillations Reflect Improving Neural Coordination Within Frontal Brain Networks During Adolescence

**DOI:** 10.3389/fnbeh.2018.00218

**Published:** 2018-09-27

**Authors:** Annika Susann Wienke, Canan Basar-Eroglu, Christina Schmiedt-Fehr, Birgit Mathes

**Affiliations:** ^1^Institute of Psychology and Cognition Research & Center of Cognitive Science, University of Bremen, Bremen, Germany; ^2^Izmir University of Economy, Izmir, Turkey

**Keywords:** maturation, adolescence, novelty, cognitive control, N2, P3a, theta oscillations, frontal brain network

## Abstract

Adolescents are easily distracted by novel items than adults. Maturation of the frontal cortex and its integration into widely distributed brain networks may result in diminishing distractibility with the transition into young adulthood. The aim of this study was to investigate maturational changes of brain activity during novelty processing. We hypothesized that during adolescence, timing and task-relevant modulation of frontal cortex network activity elicited by novelty processing improves, concurrently with increasing cognitive control abilities. A visual novelty oddball task was utilized in combination with EEG measurements to investigate brain maturation between 8–28 years of age (*n = 84*). Developmental changes of the frontal N2-P3a complex and concurrent theta oscillations (4–7 Hz) elicited by rare and unexpected novel stimuli were analyzed using regression models. N2 amplitude decreased, P3a amplitude increased, and latency of both components decreased with age. Pre-stimulus amplitude of theta oscillations decreased, while inter-trial consistency, task-related amplitude modulation and inter-site connectivity of frontal theta oscillations increased with age. Targets, intertwined in a stimulus train with regular non-targets and novels, were detected faster with increasing age. These results indicate that neural processing of novel stimuli became faster and the neural activation pattern more precise in timing and amplitude modulation. Better inter-site connectivity further implicates that frontal brain maturation leads to global neural reorganization and better integration of frontal brain activity within widely distributed brain networks. Faster target detection indicated that these maturational changes in neural activation during novelty processing may result in diminished distractibility and increased cognitive control to pursue the task.

## Introduction

Adolescence has a life-long impact on a person’s health, social inclusion and success (Pantelis et al., 2009; Blakemore and Robbins, 2012; Crone and Dahl, 2012). This age period is characterized by increased distractibility, novelty-seeking (Steinberg, 2008) and still maturing cognitive control (i.e., the ability to voluntarily guide attention, thoughts and actions, Luna et al., 2010; Blakemore and Robbins, 2012; Taylor et al., 2015). Novel situations provide learning experiences important for the adolescents’ transition into independent young adults. Distractions in dangerous situations, excessive risk- and novelty-seeking may, however, also lead to irreversible negative consequences (Steinberg, 2008; Steinberg et al., 2008). It is, thus, important to understand how successful transitions into adulthood are related to the maturing ability to maintain cognitive control in the presence of possible distracters.

Cognitive control ability during adolescence relates to the maturation of the frontal cortex and its increasing integration into long-range functional networks (Uhlhaas et al., 2009; Luna et al., 2010). Thus, understanding developmental trajectories during adolescence needs to reflect maturation of frontal brain networks.

The N2-P3a complex, an event-related response (ERP) measured by the electroencephalogram (EEG) during the novelty oddball paradigm, is suited to investigate maturation of frontal brain activation underlying cognitive control. The novelty oddball paradigm consists of a train of frequent non-targets, intertwined with rare and unexpected targets as well as un-repeated, novel stimuli. While the participant should indicate target detection by a button press, novel stimuli are not task-relevant. The frontal N2-P3a complex is elicited by unexpected and salient novels within novelty oddball paradigms. This ERP pattern seems to reflect the initial orientation response and the subsequent intentional shift of attention towards the novel stimulus (Snyder and Hillyard, 1976; Courchesne, 1978; Knight, 1984; Halgren et al., 1995; Escera et al., 1998; Demiralp et al., 2001; Friedman et al., 2001; Gumenyuk et al., 2001; Polich, 2007; Brinkman and Stauder, 2008; Bocquillon et al., 2014). The P3a is pronounced for difficult target detection, i.e., when avoiding distractions is mandatory for task performance (Polich and Comerchero, 2003; Polich, 2007). Thus, the N2-P3a complex may reflect automated and cognitive control functions handling novel distracters within the novelty oddball paradigm and, given their frontal generators (Polich, 2007; Bocquillon et al., 2011), may provide further insight into the maturation of frontal brain areas during adolescence.

Developmental studies investigating the maturing N2-P3a complex during novelty processing are scarce. Courchesne (1978) reported a large N2-like response following novels in young children (age 6–8 years) that occurred more prominently than in adults. P3a latency seems to decrease with increasing age until adulthood (Courchesne, 1978; Cycowicz et al., 1996; Oades et al., 1997; Ponton et al., 2000; but see Stige et al., 2007). Findings for the P3a amplitude are inconsistent, showing decreasing or increasing developmental trends (Courchesne, 1978; Oades et al., 1997; Kihara et al., 2010).

Measures of brain oscillations provide detailed information about selective maturational changes that may be not apparent in ERP measures. In a previous study, we could demonstrate that slow-wave amplitude decreased with increasing age, while post-stimulus amplitude modulation and timing of brain responses improved within the P3b time window (Mathes et al., 2016a,b). Connectivity measures further allow to directly investigate maturation of frontal brain network (Müller et al., 2009; Uhlhaas et al., 2009; Segalowitz et al., 2010; Ehlers et al., 2014; Hardmeier et al., 2014; Janssen et al., 2017). Thus, ERP and oscillatory brain activity were both investigated in this study.

The N2 and P3a are suggested to be dominated by frontal theta oscillations (Berns et al., 1997; Demiralp et al., 2001; Ursu et al., 2009; Hajihosseini and Holroyd, 2013). Theta oscillations (approximately 4–7 Hz) are maximal at frontal sites (Basar-Eroglu and Demiralp, 2001) and seem to drive widespread neural network activities in adults (Klimesch, 1999; von Stein and Sarnthein, 2000; Buzsáki and Draguhn, 2004; Sauseng et al., 2006; Klimesch et al., 2010; Cohen and Ridderinkhof, 2013; Lopes da Silva, 2013; Kawasaki et al., 2014). Frontal theta activity reflects cognitive control (Basar-Eroglu and Demiralp, 2001; Polich, 2007; Sauseng et al., 2010; Cahn et al., 2013; Mathes et al., 2014) and executive functions (Yordanova et al., 2004; Schmiedt-Fehr and Basar-Eroglu, 2011; Huster et al., 2013). Maturation of event-related theta oscillations indicates that in the presence of generally elevated amplitudes, task-related modulations increase with age (Müller et al., 2009; Yordanova and Kolev, 2009; Papenberg et al., 2013; Liu et al., 2014; Mathes et al., 2016a). Thus, theta oscillations are of particular importance for understanding maturational changes of fast event-related modulations during novelty processing.

The aim of the study was to investigate the frontal N2-P3a complex and concurrent theta oscillations to better understand neural maturation underlying attentional control towards distracters during the transition throughout late childhood to adolescence and early adulthood. The N2-P3a complex and concurrent theta oscillations were elicited by novel stimuli, serving as distractors within a visual novelty oddball task.

We predicted increasing dominance of the P3a in comparison to the N2 with age, thereby indicating a developmental shift from involuntary to controlled attentional resource allocation and reduced distractibility (Courchesne, 1978; Brinkman and Stauder, 2008; Bocquillon et al., 2014). We further hypothesized increased effectiveness of the post-stimulus neural activation with age, leading to decreased N2 and P3a latencies (Courchesne, 1978; Oades et al., 1997) and increased precision of task-related modulations of concurrent frontal theta oscillations. Maturational changes in frontal theta network activations may be indicated by increasing post-stimulus amplitude enhancement, inter-trial consistency and inter-site connectivity with age (Müller et al., 2009; Mathes et al., 2016a). Pre-stimulus theta amplitude was expected to decrease with age (Barry and Clarke, 2009; Cragg et al., 2011). We also hypothesized that neural maturation would be accompanied by better task performance.

## Materials and Methods

### Participants

Eighty-four volunteers participated in the study. Five participants had to be excluded due to excessive artifacts. The remaining 79 participants ranged between 8 years and 28 years (mean: 16.54, SD: 4.78, 35 males), had normal or corrected-to-normal vision, reported to be free of neurological or psychiatric diseases and pathological drug intake. Three participants reported to have reading and writing difficulties and one participant stated to have arithmetical weakness. Except for three participants, all participants older than 10 years of age were attending a secondary school aiming for university entrance degree or were university students. All except two participants were right-handed. This study was carried out in accordance with the recommendations of the ethics committee of the University of Bremen with written informed consent from all subjects. All subjects gave written informed consent in accordance with the Declaration of Helsinki. For participants below 18 years, parents also gave written consent. The protocol was approved by the ethics committee of the University of Bremen.

### Experimental Procedure

**Figure 1:**
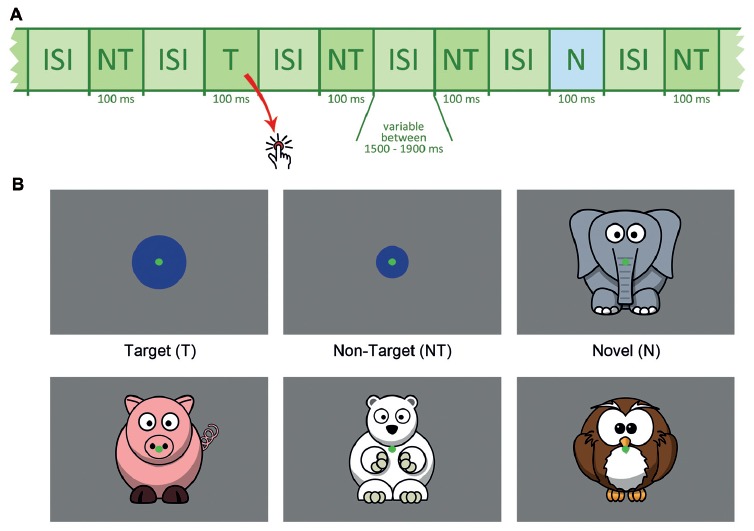
**(A)** Schematic presentation of the stimulus sequence, NT = non-target, T = target, N = novel, ISI = inter-stimulus interval. **(B)** Schematic depiction of target, non-target and novel stimuli.

Participants were seated 1.5 m in front of the monitor. Figure 1 outlines the stimulus sequence and task conditions. Two-hundred and seventy-three non-targets (NT, small blue circles), 42 targets (T, larger blue circles) and 42 novels (N, animal drawings) were presented within three runs. The appearance rate of 76% non-targets, 12% targets and 12% novels was kept equal in each run.

Target size was individually adjusted between 2.3° and 4.6° (see below). Stimulus size for non-targets (1.9°) and novels (approximately 7.6°) was fixed for all participants. The animal drawings, serving as novel distracters, were positioned in a way that capturing the novels was possible without gaze shift and that the animal’s face was near the center of the screen. Each novel was shown only once and differed considerably from all other task conditions to ensure high visual saliency. All novel stimuli were adapted from open accessible clipart from openclipart.org. To reduce eye movements, a central green dot served as the fixation point throughout the experiment.

Each stimulus was presented for 100 ms. The inter-stimulus interval (ISI) varied randomly between 1,500 and 1,900 ms (mean duration: 1,700 ms). Order of stimulus sequence was pseudo-randomized with the constraint that each target or novel was followed by a non-target. All stimuli were presented on an electromagnetically shielded 19″ raster monitor controlled by a PC with a spatial resolution of 1024 × 768 pixels and a refresh rate of 85 Hz. Subjects were asked to index each target via a button press using their right index finger.

### Individual Adjustment of Target Stimulus Size

In oddball paradigms distracters elicit a P3a-mediated orientation response only when the distinction between targets and non-targets reaches a level of difficulty that demands focused attention (Polich, 2007). Preliminary experiments of our group indicated that target detection performance in oddball paradigms increases with age. Thus, the enlargement of the target stimulus in comparison to the non-target was individually adjusted to achieve similar task demands for correctly classifying targets.

During the adjustment procedure the participant had to indicate each circle that was larger than the circle for the non-target condition with a button press. These circles had a stimulus size of either 2.3°, 3.1°, 3.9° or 4.6°. In a pseudo-randomized order 30 non-targets and 15 targets were presented within four runs. With each run target size decreased. No distracters were presented. For the EEG-Experiment the smallest target size for which detection performance reached at least 80% and the false-response rate remained below 10% was utilized as the target stimulus. This procedure achieved similar target detection rates across the age range (see “Results” section).

### EEG Measurement

EEG-Measurements took place in a dimly-lit, soundproof, electromagnetically-shielded room. A fitting electrode cap with 30 Ag-AgCl electrodes (F7, F3, Fz, F4, F8, FT7, FC3, FCz, FC4, FT8, T7, C3, Cz, C4, T8, TP7, CP3, CPz, CP4, TP8, P7, P3, Pz, P4, P8, PO3, POz, O1, O2; Easycap, Falk Minow Services) was placed onto the participant’s head according to the international 10–10 system (Chatrian et al., 1988). Linked earlobes were used as reference. Impedances were kept below 10 kΩ. Electrodes were placed above and to the right of the right eye to record the electrooculogram (EOG). The EEG was recorded at 500 Hz with band limits of 0.01–250 Hz by means of a 32-channel Brain Amp System (Brain Products^®^).

### Analysis of the Behavioral Results

Error rates and median reaction times (RTs) of correctly classified targets were determined. The coefficient of variation of RT was determined to estimate the individual trial-by-trial variability in timing of the motor response (McIntosh et al., 2008). For each measure linear regression models over the entire age range were conducted using Graphpad Prism 5.03.

### EEG Pre-processing

Analysis of the EEG data was performed in Matlab^®^. Continuous data was segmented, creating for each novel stimulus epochs ranging from 2,000 ms before to 1,998 ms after stimulus onset. Only correct trials were included in the EEG analysis, i.e., rare trials including false positive responses to novels were excluded. Epochs contaminated by eye or other artifacts between 1,000 ms before and 1,000 ms after stimulus onset were manually rejected after the recording. The minimum number of artifact-free epochs for each participant was 15. On average, 29.4 (SD: 6.1) novels were included in the analysis. A linear regression showed no indication of a relation between the individual number of included epochs and the age of participants.

### Analysis of the Stimulus-Locked N2 and P3a in the Time Domains

Artifact-free epochs were averaged for each participant. The averaged ERPs were digitally low-pass filtered with a finite impulse response at 20 Hz and baseline-corrected utilizing the mean amplitude between 700 ms and 300 ms before stimulus onset. Filtering of the ERP was performed to increase the signal-to-noise ratio by restricting the analysis to the frequency range for which significant ERP effects are normally reported.

The novelty N2 and P3a, as defined as an early anterior component of the P3 complex (Polich, 2007), were both analyzed within a region of interest (ROI) encompassing F3, Fz and F4. This ROI also matched the maximum theta response to which the analyzed ERP response is related (see below; Demiralp et al., 2001).

**Figure 2:**
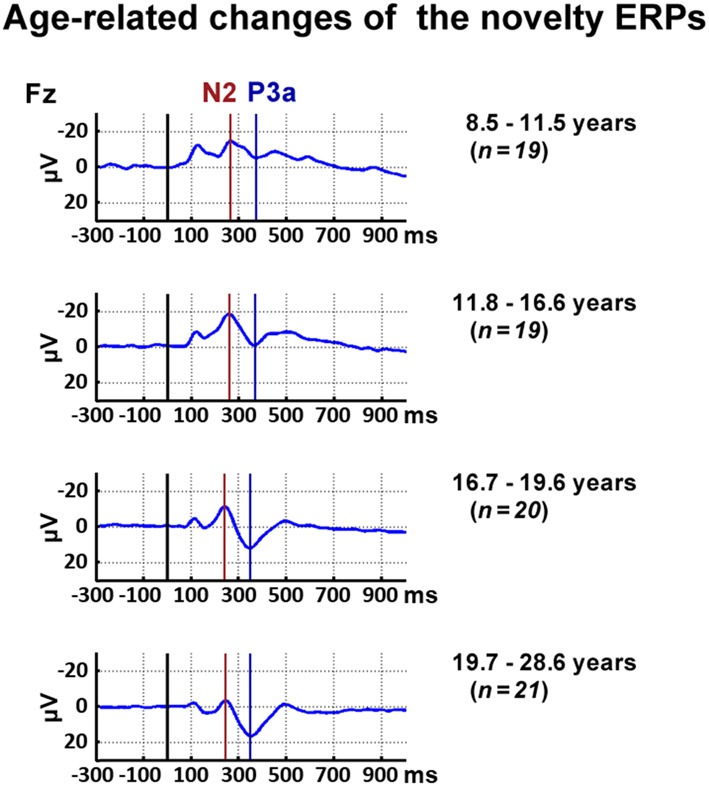
Time courses of event-related responses (ERPs) at Fz elicited by novels. For illustrational purposes, participants were divided into four age groups.

Inspection of the data indicated, in accordance with the literature, a decrease of the latency of the novelty N2 and the P3a with age. Thus, the N2 and P3a latency were analyzed for the frontal ROI as the maximum negative peak between 200 ms and 500 ms and the maximum positive peak 300–500 ms post-stimulus averaged, respectively. To reflect the latency shift, the N2 mean amplitude was analyzed between 210 ms and 310 ms post-stimulus for all participants below 16.6 years and between 190 ms and 290 ms post-stimulus for all participants above 16.6 years. In accordance, the P3a mean amplitude was analyzed between 400 ms and 500 ms post-stimulus for all participants below 13.5 years and between 300 ms and 400 ms post-stimulus for all participants above 13.5 years. Figure 2 displays ERP courses for participants divided into four age groups to illustrate the chosen time windows.

### Time-Frequency Transformation Extracting Oscillatory Dynamics in the Theta Band

Theta time–frequency analysis was conducted in an analogous manner to previous studies of our group (Mathes et al., 2012, 2014, 2016a,b). All included epochs were transformed with a single Morlet wavelet of five cycles and a center frequency of 5.5 Hz utilizing the toolbox of Torrence and Compo (1998). This wavelet, as defined by one standard deviation, covered the theta frequency range between 4.4 Hz and 6.7 Hz. The approximate length of the wavelet was 500 ms, with the contribution decreasing with increasing time distance from the analyzed time window according to the Gaussian shape of the Morlet wavelet. The time width was estimated as twice the folding time of the used Morlet wavelet, that is, the time after which the Gaussian window has dropped to exp(−2) ≈ 14% (for details see Torrence and Compo, 1998). The wavelets were normalized to have unity energy. In order to enhance comparability with signal amplitude if calculated by a Fourier transform, the transformed data was multiplied by the square-root of the sampling interval (Torrence and Compo, 1998; Mathes et al., 2014).

### Determination of Time Windows and ROIs for Frontal Theta Band Analysis

#### Pre-stimulus Single-Trial Amplitude

The amplitude AMP was determined by calculating the average of the absolute value of the transformed data of each included single-trial. Age-related differences in baseline brain activity were estimated by theta amplitudes between 700 ms and 300 ms preceding stimulus onset. Mean amplitude values over the baseline period were pooled together in a frontal ROI (Fz, FCz, Cz) incorporating the maximum theta response.

All other measures were determined for the post-stimulus period to reflect task-related changes of theta oscillations occurring concurrently with the N2 and P3a. These measures were analyzed between 200 ms and 400 ms post-stimulus after visual inspection of the grand average and all individual subject responses confirmed that the maximum of post-stimulus modulations occurred in this time window disregarding of age.

#### Post-stimulus Amplitude Modulation

To estimate amplitude changes induced by the stimulus onset, the mean baseline log spectrum was subtracted from each spectral estimate, producing a baseline-normalized time–frequency distribution. The estimated values indicate amplification or attenuation (in dB) at a given latency relative to the baseline (see Delorme and Makeig, 2004 for a detailed description of the method). The chosen baseline between 300 ms and 700 ms before stimulus onset is due to the width in time of the wavelets assessed for filtering (for more information see Mathes et al., 2012, 2014). To reflect the topographical distribution of the maximal post-stimulus amplitude modulations, the ROI utilized for the statistical analysis included the electrode sites Fz, FC3, FCz, FC4, and Cz.

#### Inter-trial Phase Coherence (ITC)

Inter-trial phase coherence (ITC) allows the estimation of phase consistency over trials within a particular time–frequency window, i.e., the phase-locking with respect to an experimental event, e.g., stimulus onset. A value of ITC = 0 represents the absence of a consistent EEG phase; values near 1 indicate perfect alignment (see Delorme and Makeig, 2004 for a detailed description of the method). The frontal ROI utilized for statistical analysis matched the post-stimulus amplitude modulation.

#### Functional Brain Connectivity by the Weighted Phase Locking Index (wPLI)

Functional brain connectivity specifies synchronous neural activity between different brain regions. A multitude of metrics with different pros and cons exists. The weighted phase locking index (wPLI) estimates inter-site phase coherence, based on the imaginary component of the cross-spectrum i.e., the phase difference information of the oscillatory brain responses (Vinck et al., 2011). This measure is suitable to investigate moment-to-moment variability of functional brain connectivity without being distorted by spurious volume conduction effects (Cohen, 2014; Bastos and Schoffelen, 2015). The wPLI does, contrary to other measures, not require an age-fitting brain model. Further, the wPLI is suitable for data-driven analysis (Cohen, 2014). These properties make the wPLI specifically suitable for studying developmental changes in event-related functional brain connectivity.

Connectivity strengths of 48 electrode-pairs were averaged between 200 ms and 400 ms post-stimulus and analyzed as the amplification or attenuation relative to the baseline. Post-stimulus wPLI estimates were subtracted from the average baseline wPLI and, to control for signal variations, the result was subsequently divided by the standard deviation of the average baseline wPLI (see also Uhlhaas et al., 2006). Electrode pairs were selected to focus on maturation of frontal brain networks. Electrode sites Fz, FCz, and Cz were defined as seed electrodes. Electrode pairs were grouped into either belonging to short-range fronto-central connections (FC, including electrode pairs between one of the seed electrode sites and F3, F4, FC3, FC4, C3 and C4, respectively) or to long-range anterior-posterior connections (AP, including electrode pairs between one of the seed electrode sites and P3, Pz, P4, PO3, POz, PO4, O1 and O2, respectively). Visual inspection confirmed that the chosen electrode pairs and time window contained the maximum wPLI values for the frontal seeds to all other electrode sites.

### Statistical Regression Analysis of EEG-Data

Regression analyses of age-related changes in EEG measures were conducted separately using Graphpad Prism 5.03.

A linear model was fitted to the data and compared to a linear model with slope zero, i.e., the model assuming no age-related change. A significant difference between both models, thus, indicated an age-related linear change in the data. If this was the case, the additional benefit of using a higher-order quadratic instead of a linear regression model was tested (see Brown et al., 1983; van Dinteren et al., 2014a,b; for discussion and similar procedure). This was done by the extra-sum-of-squares *F*-test (implemented in Graphpad Prism). This test takes into account that although any model containing more parameters (and therefore more degrees of freedom) improves data description, this improvement may not overcome the disadvantage of adding unnecessary complexity (i.e., overfitting of the model). Thus, the test compares differences of the sum-of-squares between both linear models and the quadratic model while controlling for the number of parameters (and therefore different numbers of degrees of freedom). The null hypothesis assumes that the simpler model (i.e., the linear model) fits the data more appropriately.

This statistical procedure takes into account that developmental changes may not be simply linear but may slow down or accelerate in specific age ranges (see also van Dinteren et al., 2014a,b).

In the results section, each data set is presented by a scatter plot over age in combination with the best-fit regression model. Linear curves are described by their slope (B1) and intercept (B0), while quadratic curves are described by their quadratic coefficient (B2), their linear coefficient (B1) and their constant term (B0) and the peak of the quadratic curve.

For all statistical tests was the significance level set to *p* < 0.05.

## Results

**Figure 3:**
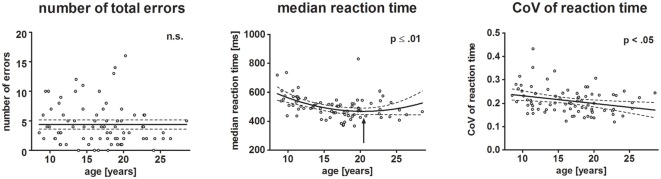
Scatterplots depict behavioral results. Each dot represents one participant. Left, middle and right plot represent the number of total errors, median reaction time (RT) and individual variation of RTs, respectively. The ordinates represent age (years). Solid lines show the estimation of age-related changes according to the best-fit regression model (linear or quadratic). Broken lines indicate the 95% confidence interval of the model. Arrows indicate the minimum estimated by the quadratic regression model.

**Figure 4:**
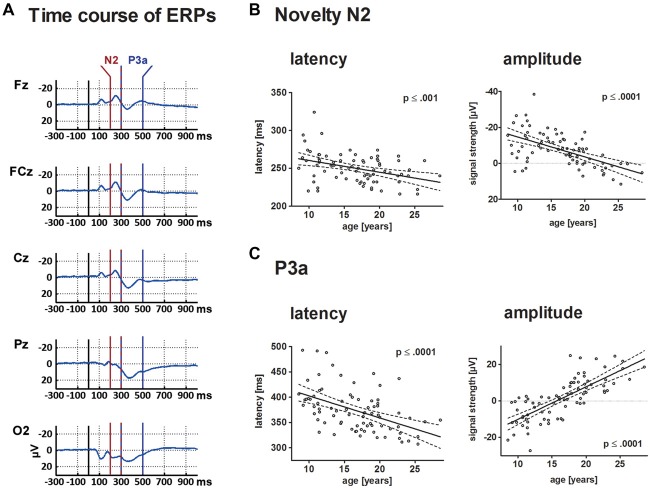
This depicts the ERP elicited by novels at central electrode sites **(A)** and individual results for N2 and P3a in a scatterplot **(B,C)**. For both components, results are depicted in a scatterplot as a function of age and either latency or mean amplitude. Each dot represents one participant. The ordinates represent age (years). Solid lines show the estimation of age-related changes according to the best-fit regression model (either linear or quadratic). Broken lines indicate the 95% confidence interval of the model. Time windows for the analysis are detailed in the “Materials and Methods” section.

**Figure 5:**
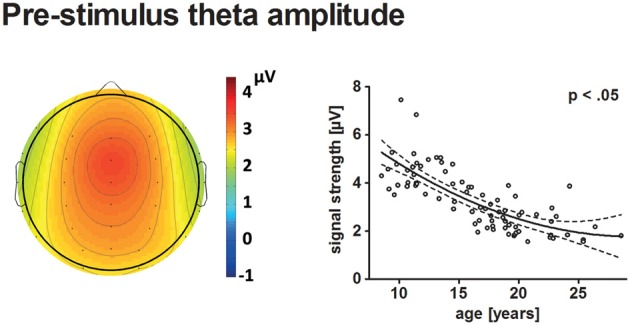
The topographical distribution of pre-stimulus theta amplitudes across all participants is illustrated on the left. The scatterplot on the right indicates the age-related decrease of pre-stimulus theta amplitudes (see Figure 4 for further information on scatterplots).

**Figure 6:**
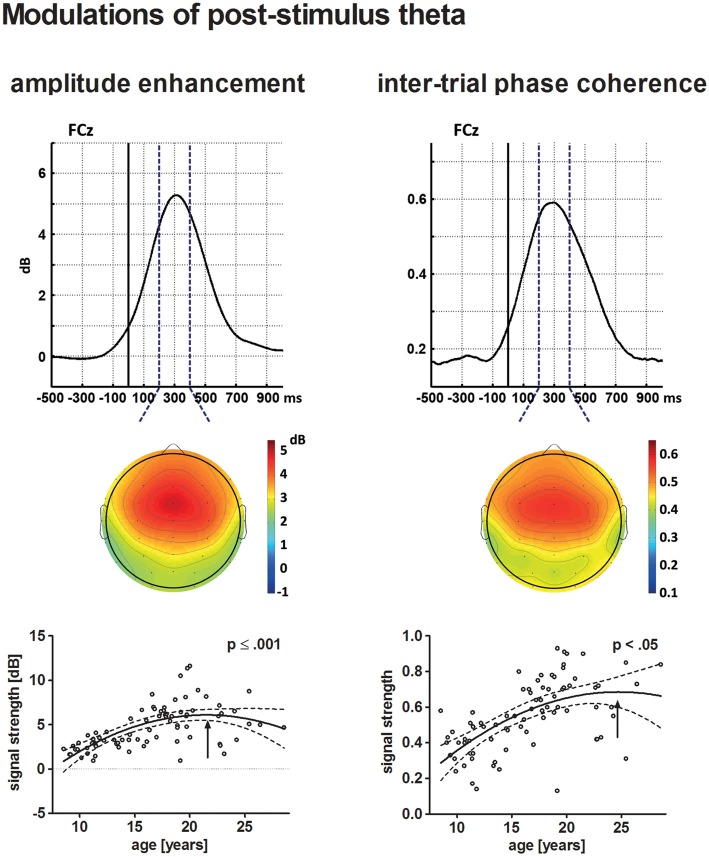
The upper row depicts the averaged time courses at the electrode site FCz for theta post-stimulus amplitude enhancement (left) and tnter-trial phase coherence (ITC; right) across all participants. Topographical distributions within the time–frequency window defined for the statistical analysis (broken lines) are displayed underneath. The solid vertical line indicates stimulus onset. The lower row depicts the age-related increase of post-stimulus amplitude enhancement (left) and ITC (right) in a scatterplot (see Figure 4 for further information on scatterplots).

**Figure 7:**
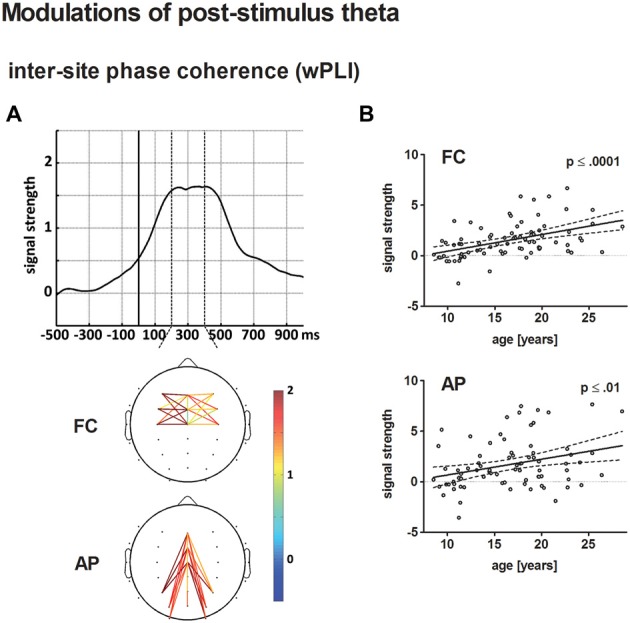
**(A)** The top displays the averaged time course of baseline-corrected weighted phase locking index (wPLI) estimates across all electrode pairs and participants entering the statistical analysis. The solid vertical line indicates stimulus onset. The broken vertical lines indicate the time window for statistical analysis. The bottom illustrates the grand-averaged, baseline-corrected wPLI estimate for fronto-central (FC) and anterior-posterior (AP) electrode pairs according to the statistical analysis. Panel **(B)** depicts the age-related increase of post-stimulus wPLI estimates for FC (top) and AP electrode pairs (bottom) in a scatterplot (see Figure 4 for further information on scatterplots).

Figure 3 displays the behavioral results. The ERPs and statistical results for the novelty N2 and P3a are displayed in Figures 2, 4. Topographical distribution of the theta response and scatter plots illustrating developmental trajectories are depicted in Figure 5 for pre-stimulus amplitude, in Figure 6 for post-stimulus amplitude modulation and ITC, and in Figure 7 for wPLI estimates. Tables 1 and 2 summarize the best-fit regression models and their statistical results for behavioral and electrophysiological measures.

**Table 1:** Description of best-fit regression models for number of epochs and behavioral data of the novelty oddball task.

	Model	*F*-value (df)	B0	B1	B2	R^2^
Number of epochs	L	(1,77) 0.4	29.4	0.0	-	0.0
Error rate	L	(1,77) 0.2	4.4	0.0	-	0.0
Median RT	Q	(1,76) 7.2**	828.4	−35.3	0.9	0.1947
CoV RT	L	(1,77) 6.8*	0.3	−0.003	-	0.0810

*p < 0.05, **p ≤ 0.01.

**Table 2:** Description of best-fit regression models for the P3a and N2 response components and the correlated theta response during novelty processing.

Component	Characteristics		Model	*F*-value (df)	B0	B1	B2	R^2^
P3a	Latency		L	(1,77) 24.1****	445.9	−4.3	-	0.2384
	Amplitude		L	(1,77) 103.5****	−27.9	1.8	-	0.5733
N2	Latency		L	(1,77) 13.0****	275.8	−1.6	-	0.1452
	Amplitude		L	(1,77) 37.0****	−25.8	1.1	-	0.3243
Theta	Pre-stimulus amplitude		Q	(1,76) 4.9*	8.7	−0.5	0.008	0.6024
	Post-stimulus amplitude enhancement		Q	(1,76) 13.4***	−8.3	1.3	−0.031	0.3984
	ITC		Q	(1,76) 4.7*	−0.2	0.1	−0.002	0.3633
	wPLI	FC	L	(1,77) 20.6****	−1.2	0.2	-	0.2107
		AP	L	(1,77) 8.2**	−0.9	0.2	-	0.0958

*p < 0.05, **p ≤ 0.01, ***p ≤ 0.001, ****p ≤ 0.0001.

### Behavioral Results

Neither the total number of errors (mean: 5.48%, SD: 5.85), nor omission (mean: 2.38, SD: 2.55) and commission (mean: 1.88, SD: 1.83) errors varied with age. Median reaction time (mean: 517.54 ms, SD: 86.4 ms) decreased with increasing age. The non-Gaussian distribution of the residuals of the regression analysis indicated that the exact age-related pattern cannot be precisely determined. The best-fit regression model indicated a quadratic trend with the decrease of RT slowing until it reached a minimum at 20.6 years of age (*p* ≤ 0.01).

### Mean Amplitude and Latency of the N2 and P3a

#### N2

Mean amplitude and latency of the frontal N2 decreased with increasing age. Age-related changes of the N2 amplitude and N2 latency are described optimally by a linear decrease with age (*p* ≤ 0.001 and *p* ≤ 0.0001, respectively). Mean latency of the N2 peak at FCz was 250 ms (SD: 19.5).

#### P3a

The frontal P3a mean amplitude increased and P3a latency decreased linearly with age (*p* ≤ 0.0001 for both comparisons). Mean latency of the P3a peak at FCz was 374 ms (SD: 42.6).

### Time-Frequency Analysis of Theta Oscillations

#### Pre-stimulus Amplitude

Pre-stimulus theta amplitudes had a frontal maximum and decreased with age (*p* < 0.05). The best-fit model indicated a quadratic trajectory, i.e., the reduction of pre-stimulus amplitude with age becomes less apparent for older participants. A minimum might be reached during young adulthood at approximately 29.7 years.

#### Post-stimulus Amplitude Enhancement

The individual maximum of post-stimulus theta amplitude enhancement at FCz occurred on average 310 ms following stimulus onset. Post-stimulus amplitude enhancement elicited by novel stimuli increased with increasing age. The best-fit regression model indicated a quadratic trajectory, i.e., the pattern of increasing amplitude enhancement is more prominent for younger participants and becomes less apparent until it reaches a maximum during young adulthood (approximately 21.5 years of age, *p* ≤ 0.001).

#### ITC

Maximum theta ITC was observed at the electrode site FCz 300 ms after stimulus onset. Theta ITC increased with increasing age, but becomes less apparent for older participants. A maximum might be reached during young adulthood at approximately 24.7 years of age. This was indicated by the best-fit quadratic regression analysis (*p* < 0.05).

#### wPLI

Connectivity strength, averaged for FC and AP connections, increased as a broad peak from approximately 220–420 ms post-stimulus. Both, baseline-corrected FC and AP theta phase coupling increased linearly with age (*p* < 0.0001 and *p* ≤ 0.01, respectively). A direct comparison of the best-fit regression models for FC and AP phase coupling revealed no significant differences.

## Discussion

We investigated maturational changes of the frontal N2-P3 complex and concurrent theta oscillations during novelty processing in the 8–28 years age range. The aim of the study was to better understand improvements in cognitive control functions and related changes in frontal brain network functioning during adolescence. Frontal N2 amplitude decreased and P3a amplitude increased with age. Latency of both ERPs decreased during development. In the presence of increased pre- stimulus theta amplitudes, post-stimulus modulations of frontal theta oscillations are diminished before the transition into young adulthood. Detailed analysis revealed that developmental changes during late childhood and adolescence affect post-stimulus amplitude enhancement, temporal precision and inter-site connectivity of frontal theta oscillations.

### Task Performance

Individual adaptation of task difficulty ensured comparable detection rates of targets across the age range. Decreasing RT with age indicate that even after controlling detection rates children need more processing time than adults to indicate target detection. The age-related decrease of RT slows down during maturation, indicating that adolescents nearly reach adult levels but late brain maturation still improves RT. Disregarding of speed does the timing of motor responses stabilizes during the course of development. This is indexed by a linear decrease of RT variability (CoV) measures with age. The unchanged improvement in RT variability during the transition between childhood, adolescence and young adulthood indicates the importance of trial-by-trial measures to understand late brain maturation. These findings are in line with a variety of studies and may result from reduced distractibility and increased attentional control to pursue the task (Blakemore and Choudhury, 2006; Luna et al., 2010; Blakemore and Robbins, 2012; Taylor et al., 2015).

### Maturation of the N2-P3a Complex

The interrelation of the frontal N2 and P3a during development has only been rarely investigated. Studies reported that for children the P3a, and for young adults the N2 was not reliably detected in individual ERPs (Courchesne, 1983; Oades et al., 1997; Segalowitz and Davies, 2004). Our results mark this observation as a decrease of the N2 and an increase of the P3a amplitude with age. These developmental changes seem accompanied by faster neural information processing, as indicated by decreasing latencies of the N2 and P3a, and related to faster motor reaction to targets. While the increase in neuronal processing speed and motor reactions may slow down during adolescence, recruitment of neuronal circuits, as indicated by ERP amplitudes, seems to increase continually until young adulthood is reached.

Children pay more attention to task-irrelevant distractors (Segalowitz and Davies, 2004; Wetzel et al., 2006, 2016). Diminished amplitudes of the N2 with ongoing development might reflect a reduction of the involuntary orientation response towards the novel distracter and better attentional control (Snyder and Hillyard, 1976; Knight, 1984; Halgren et al., 1995; Friedman et al., 2001; Bocquillon et al., 2014). Increasing P3a amplitude with age may be related to improving abilities to inhibit attentional shifts towards the distracter or to disengage attention from distractors during early stimulus processing (Courchesne, 1978; Escera et al., 1998; Friedman et al., 2001; Gumenyuk et al., 2001; Wetzel et al., 2006; Polich, 2007; Lackner et al., 2013). The increasing dominance of the P3a and faster neural processing with ongoing brain maturation indicates improved attentional control of information processing following novel distracters.

The novelty N2 is associated with the anterior cingulate cortex (Kropotov et al., 2011; Wessel et al., 2012; Bocquillon et al., 2014) and the P3a with the prefrontal cortex (Knight, 1984; Cycowicz and Friedman, 1997; Daffner et al., 2003; Folstein and van Petten, 2008). Thus, developmental changes of the N2-P3a complex may reflect immaturity of the frontal cortex (e.g., Cycowicz et al., 1996; Čeponienė et al., 2004; Flores et al., 2010).

It is important to note that not all studies report a developmental increase of the P3a amplitude (e.g., Courchesne, 1978, 1983; Cycowicz and Friedman, 1997; Oades et al., 1997; Stige et al., 2007; Kihara et al., 2010). Reasons for this may be multifold. Variations in task design might account for different findings (Courchesne, 1978; Cycowicz et al., 1996; Conroy and Polich, 2007). Superposition of the N2 and P3a may vary for different age groups and obscure developmental trajectories for both components (see Oades et al., 1997).

The N2-P3a complex is dominated by the theta response (Demiralp et al., 2001; Isler et al., 2008; Müller et al., 2009; Hajihosseini and Holroyd, 2013; Prada et al., 2014). Thus, investigation of theta oscillations might help resolving controversies about developmental trajectories underlying ERP amplitude measures (see also Mathes et al., 2016a).

### Maturation of the Frontal Theta Response

Brain oscillations have been linked to the general capacity of the brain to coordinate neural information processes between segregated, functionally distinct brain areas into a regulated time-flow of activation within neural networks and, thereby, enable highly organized brain states necessary for perception, cognition and action (Basar and Güntekin, 2009; Buzsáki et al., 2013). Oscillatory theta activity allows neural information transfers over large distances (von Stein and Sarnthein, 2000; Lopes da Silva, 2013). In healthy adults, anterior theta may be influencing neural activity of posterior brain sites (Sauseng et al., 2006; de Borst et al., 2012; Lee and D’Esposito, 2012; Cohen and van Gaal, 2013). These characteristics of frontal theta networks are important for a wide range of cognitive processes (Buzsáki, 2005), most prominently attentional control and response inhibition (Klimesch et al., 2010; Sauseng et al., 2010; Cohen and Cavanagh, 2011; Schmiedt-Fehr and Basar-Eroglu, 2011; Mathes et al., 2014; Müller et al., 2017). In schizophrenia, a neuropsychiatric illness linked to adolescent development (Pantelis et al., 2009) and diminished cognitive control (Basar-Eroglu et al., 2007), disturbed modulation of event-related theta oscillations have been repeatedly reported (Schmiedt et al., 2005; Basar-Eroglu et al., 2008; Bates et al., 2009; Mathes et al., 2016b; Javitt et al., 2018). Thus, our results on developmental changes of theta oscillations relate to the maturing integration of the frontal cortex within widely distributed networks, maturing cognitive control functions, and fundamental health risks during adolescence.

The finding of decreasing pre-stimulus theta amplitude throughout adolescence confirms previous studies (Mathes et al., 2016a; Barry and Clarke, 2009; Yordanova and Kolev, 2009). In conjunction with the finding of increasing post-stimulus enhancement of theta amplitudes, our results indicate downregulation of ongoing theta activity in favor of transient, task-related adaptations of oscillatory activity. During maturation post-stimulus amplitude does not only increase, the periodic characteristic (the phase) of the oscillatory theta response stabilizes with respect to the occurrence of an event, as indicated by ITC, and with respect to the activation pattern between electrode sites, as indicated by wPLI. Thus, our results indicate increasing efficiency and timing accuracy of functional theta networks underlying the N2-P3a complex elicited by novelty processing.

Similarly Müller et al. (2009) reported a general increase of slow-wave ITC for underage compared to young, adult participants during novelty processing. Using an emotion-regulation task, Zhang et al. (2013) have shown that the post-stimulus amplitude increase of anterior theta reflects distraction and increases during adolescence. The finding of increasing post-stimulus modulations of the frontal theta response with age are also in line with other developmental studies on theta oscillations (Yordanova and Kolev, 1997b; Michels et al., 2012; Liu et al., 2014; Chorlian et al., 2015; Mathes et al., 2016a), and may generalize over a broad frequency range (Werkle-Bergner et al., 2009; Uhlhaas and Singer, 2011) However, maturational trajectories for evoked theta power may be different (Müller et al., 2009; Corcoran et al., 2018). In adolescents, improved precision of timing in low frequency oscillations may have a direct impact on behavioral performance (Papenberg et al., 2013; Liu et al., 2014; Bender et al., 2015).

### Maturation of Frontal Brain Networks

Frontal cortex maturation is often highlighted in studies about adolescence (Naghavi and Nyberg, 2005; Luna et al., 2010; Cole et al., 2014). Maturation of the neural architecture, necessary for efficient information transfer, i.e., neuronal myelination and pruning, lead to considerable changes in frontal brain regions during adolescence (Giedd et al., 1999; Giedd, 2004; Paus, 2010). These changes cannot be fully understood by focusing on the frontal cortex only. Our results of increasing inter-site connectivity within FC and AP networks implicate that maturation during adolescence increases integration of frontal brain activity within widely distributed brain networks. Maturation during adolescence seems to trigger global neural reorganization (Whitford et al., 2007; Uhlhaas and Singer, 2011).

Development during adolescence might be characterized by the maturing control-function of the frontal cortex coordinating neural information flow within distributed cortical networks (Sauseng et al., 2006; Luna et al., 2010; de Borst et al., 2012; Lee and D’Esposito, 2012; Cohen and van Gaal, 2013). Efficient adaptions of transient neural activity within frontal brain networks may relate to the broad range of higher cognitive abilities that still improve during adolescence (Blakemore and Choudhury, 2006; Luna et al., 2010). Understanding of neural development will profit from studies directly relating maturation of oscillatory networks to the structural architecture (see, Casey et al., 2000; Segalowitz et al., 2010; Sui et al., 2014 for reviews) and neurochemical pathways of the brain (Koch et al., 2016; Haenschel and Linden, 2011).

Our results should also sensitize for differential developmental trajectories in different measures of the oscillatory activity (see also Nanova et al., 2011). The decrease in pre-stimulus amplitudes, increases in post-stimulus amplitude enhancement, and inter-trial consistency for theta slow down with increasing age. The increase of inter-site connectivity continues linearly until young adulthood is reached. This indicates the ongoing impact of maturing frontal brain networks for adolescents during their transition into young adulthood. Future studies are needed to confirm differential developmental trajectories underlying neural activation pattern and its relation to ERP measures.

### Limitations

The current analysis reflected not only on the direction of maturational changes in brain measures but also indicated if with ongoing maturation these changes became less apparent or not. This study covered general developmental patterns over a broad age range between late childhood, adolescence and young adulthood. ERP and oscillatory measures may, however, also be utilized to index developmental changes in steps of 1 or 2 years of age (Yordanova and Kolev, 1997a; Ponton et al., 2000; Uhlhaas et al., 2009; Miskovic et al., 2015).

Future studies are needed to verify the current results within a longitudinal design, since differences between participants may cloud the observation of developmental changes. For example, although recruitment strategies aimed at similar educational opportunities across the age-range, the reached educational level as grown-ups cannot be controlled for in a cross-sectional design.

## Conclusion

Taken together, developmental changes of the frontal N2-P3a complex and concurrent event-related theta oscillations reflect maturation within widely distributed frontal brain networks. With the transition from late childhood to adolescence and young adulthood neural processing of novel stimuli becomes faster and the neural activation pattern more precise in timing and amplitude modulation. Faster target detection indicated that these maturational changes in neural activation during novelty processing may result in diminished distractibility and increased cognitive control to pursue the task. This study also underlines that investigation of neural oscillations help to understand maturational changes not apparent in ERP measurements.

## Data Availability

The raw data supporting the conclusions of this manuscript will be made available by the authors, without undue reservation, to any qualified researcher.

## Author Contributions

ASW, CB-E and BM designed the study. ASW and BM ran the measurements and data analysis. All authors, ASW, CB-E, CS-F, and BM cooperated in preparing the manuscript.

## Conflict of Interest Statement

The authors declare that the research was conducted in the absence of any commercial or financial relationships that could be construed as a potential conflict of interest. The handling Editor declared a past co-authorship with one of the authors CB-E.
